# A Modified Implementation of Tristate Inverter Based Static Master-Slave Flip-Flop with Improved Power-Delay-Area Product

**DOI:** 10.1155/2014/453675

**Published:** 2014-02-27

**Authors:** Kunwar Singh, Satish Chandra Tiwari, Maneesha Gupta

**Affiliations:** ^1^Department of Electrical Engineering, Delhi Technological University, Room No. FW1-SF1, EED, DTU, New Delhi 110042, India; ^2^Division of ECE, Netaji Subhas Institute of Technology (NSIT), University of Delhi, Sector 3, Dwarka, New Delhi 110078, India

## Abstract

The paper introduces novel architectures for implementation of fully static master-slave flip-flops for low power, high performance, and high density. Based on the proposed structure, traditional C^2^MOS latch (tristate inverter/clocked inverter) based flip-flop is implemented with fewer transistors. The modified C^2^MOS based flip-flop designs mC^2^MOSff1 and mC^2^MOSff2 are realized using only sixteen transistors each while the number of clocked transistors is also reduced in case of mC^2^MOSff1. Postlayout simulations indicate that mC^2^MOSff1 flip-flop shows 12.4% improvement in PDAP (power-delay-area product) when compared with transmission gate flip-flop (TGFF) at 16X capacitive load which is considered to be the best design alternative among the conventional master-slave flip-flops. To validate the correct behaviour of the proposed design, an eight bit asynchronous counter is designed to layout level. LVS and parasitic extraction were carried out on Calibre, whereas layouts were implemented using IC station (Mentor Graphics). HSPICE simulations were used to characterize the transient response of the flip-flop designs in a 180 nm/1.8 V CMOS technology. Simulations were also performed at 130 nm, 90 nm, and 65 nm to reveal the scalability of both the designs at modern process nodes.

## 1. Introduction

Flip-flops are the key elements used in sequential digital systems. The appropriate selection of flip-flop topologies is instrumental in the design of VLSI integrated circuits such as microprocessors, microcontrollers, and other high complexity chips. However, factors such as high performance, low power, transistor count, clock load, design robustness, power-delay, and power-area tradeoffs are generally considered before choosing a particular flip-flop design. The highest operating frequency of clocked digital systems is determined by the flip-flops. Flip-flops and clock distribution network generally account for 30–70% of the total chip power consumption [1, 2]. Clock load is another major concern for digital system designers and several contributions have been reported in the past to reduce clock load and the associated power dissipation in the clocking network [3–5]. A design with elevated transistor count occupies a larger area on chip and leads to an increase in the overall manufacturing cost. Hence, design and implementation of low power high performance flip-flops with the least possible chip area is the main target of the modern chip manufacturing industry.

Flip-flops are broadly classified into three main categories, namely, master-slave [6–11], pulse triggered [12–17], and differential flip-flops [18–21]. Among them, master-slave and pulse-triggered flip-flops are the most efficient in terms of power-delay product. Master-slave flip-flops exhibit positive (negative) set-up time (hold time) requirements and hence not suitable for high speed systems due to extended data to output delays. But they are power efficient and can be used in low power applications. However, their main limitation is less robustness to clock skew. Pulse-triggered flip-flops have negative set-up time and thus lead to smaller data to output delay. They exhibit inherent soft clock edge property which minimizes clock skew related cycle time loss.

**Figure 1:**
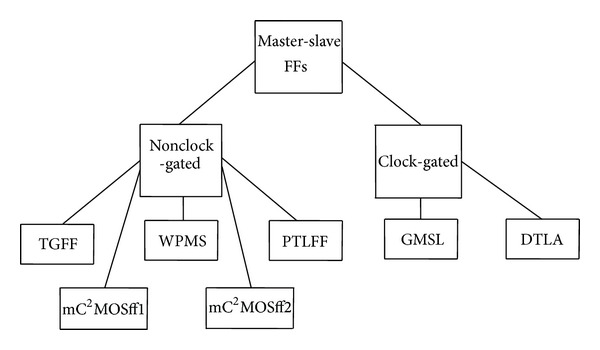
Classification of master-slave flip-flops.

A classification of master-slave flip-flops is further elaborated in Figure 1. Clock-gated topologies exhibit internal clock gating to suppress the power consumption at lower data switching activities based on a clock gating logic and a comparator circuit. However, clock gated flip-flops have extended latency due to enhanced clock to output delays along with increased chip area overhead. Clock gated structures generally consume lesser power at low switching activities [22]. TGFF represents the best choice in the nonclock gated flip-flop category in terms of power-delay product [6], whereas existence of NMOS transistors in the critical path along with partially nongated keepers leads to less significant power-delay tradeoff characteristics in case of write port master-slave flip-flop (WPMS) [7, 8] and pass transistor logic based flip-flop (PTLFF) [9].

In this paper, we introduce an alternative design approach for designing C^2^MOS based master-slave flip-flop, based on a new architecture with reduced transistor count and improved power-delay-area product. The proposed configurations mC^2^MOSff1 and mC^2^MOSff2 fall under the nonclock gated flip-flop category as shown in Figure 1.

The rest of the paper is organized as follows.  Section 2 compares the conventional master-slave flip-flop configurations with proposed designs.  Section 3 highlights the simulation parameters and test bench along with techniques used for transistor sizing and methodology adopted for optimization of timing and power-delay product.  Section 4 describes the simulation results.  Section 5 concludes the paper. An appendix is added to show calibration of parameters for delay calculations using LE theory and to outline the strategy followed for designing the eight-bit ripple counter.

## 2. Overview of Previous Work and Proposed Designs

**Figure 2:**
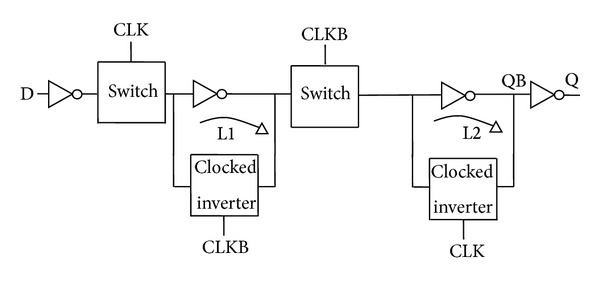
Conventional architecture with clocked switches in the critical path.

Figure 2 shows the conventional master-slave flip-flop architecture, whereby two regenerative loops (L1 and L2) are present in the master and slave sections to account for a static functionality. Both loops operate independently of each other on complementary clock signals. Regenerative loops are composed of cross coupled inverters. It can be observed from Figure 2 that for each loop, regenerative action is achieved through one inversion in the forward (critical) path while the other (clocked) inversion takes place in the feedback path. Moreover, there is no common component between both loops.

**Figure 3:**
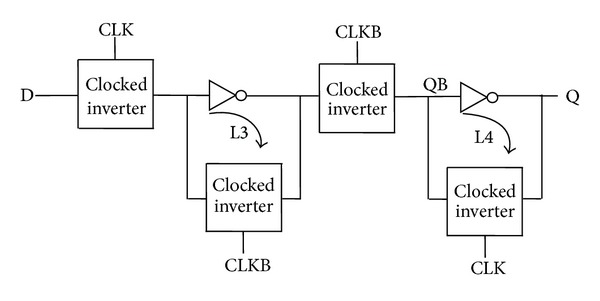
Conventional architecture with clocked inverters in the critical path.

Since an inverter followed by transmission gate is equivalent to a clocked inverter, the combination is replaced by a clocked inverter to form a C^2^MOS based flip-flop architecture as shown in Figure 3 [23]. Two regenerative loops L3 and L4 are used in a similar manner as in the previous case to maintain the static nature of the flip-flop.

**Figure 4:**
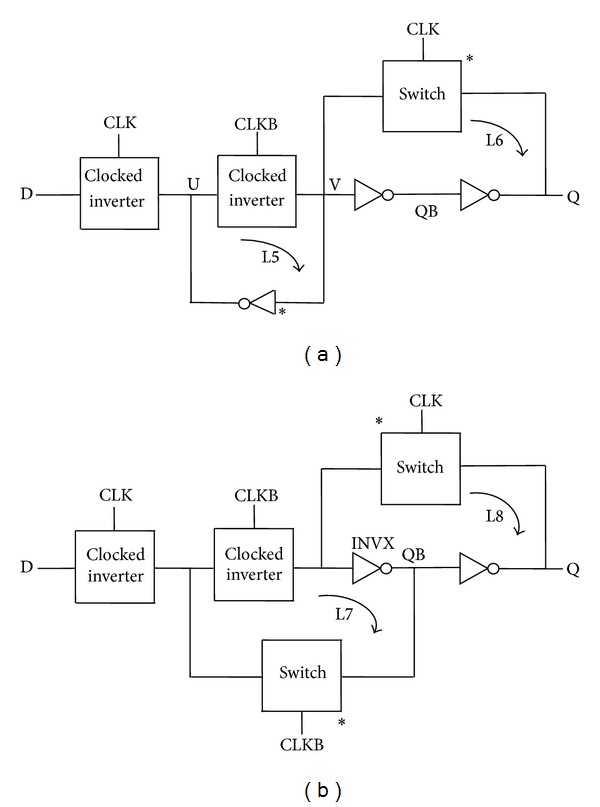
Proposed architectures.

However, in the proposed architecture as reported in Figure 4(a), both inversions take place in the forward (critical) path and the loop is completed by a clocked switch for loop L6 while loop L5 is completed by using an inverter in the feedback path. It is clearly noticed from Figure 4(a) that the output node is always driven and never floating thus ensuring a static flip-flop operation. The size of transistors in the feedback path marked by asterisks (∗) is kept at 360 nm (minimum technology width) to eliminate race conditions at nodes U and V. Yet another implementation is shown in Figure 4(b) which uses inverter INVX in the critical path and a clocked switch to form a regenerative loop L7. It is to be noted that INVX is common to both the regenerative loops L7 and L8 which is contrary to the realization of previous architectures.

**Figure 5:**
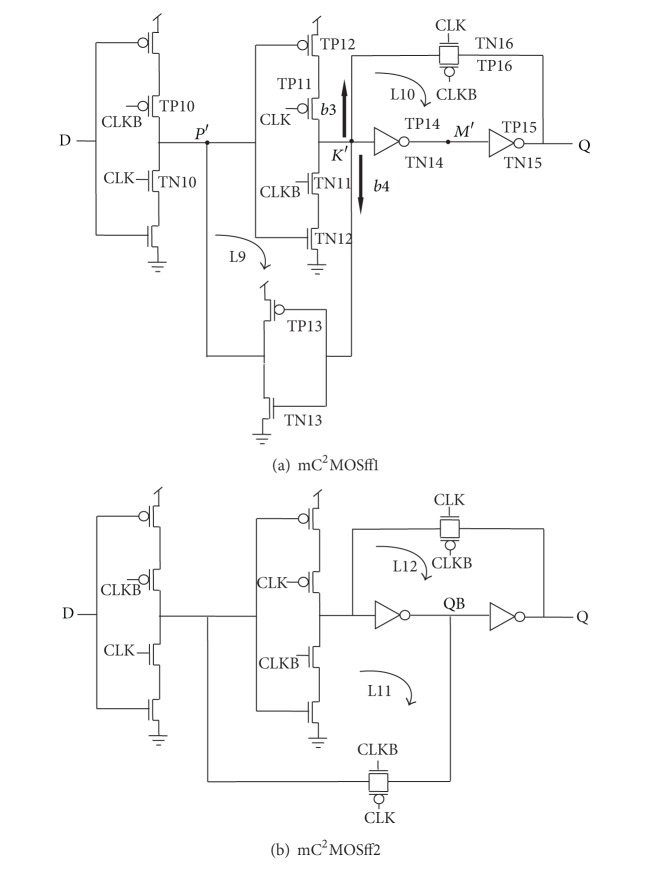
Schematic diagrams of the proposed designs.

**Figure 6:**
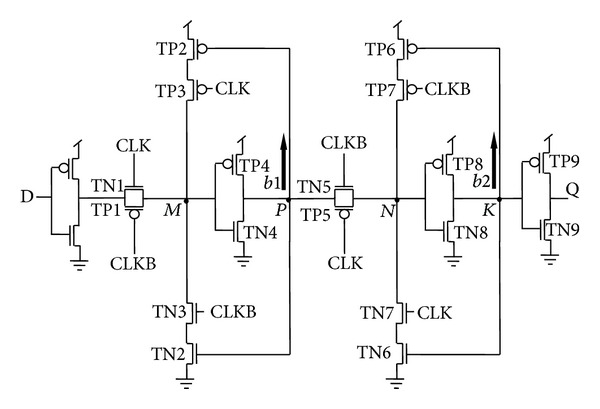
TGFF based on conventional architecture.

Figure 5 represents the actual circuit design based on the proposed architectures in Figure 4, while TGFF is implemented using transmission gates as switches in the conventional architecture as demonstrated in Figure 6.

It can be clearly observed that mC^2^MOSff1 and mC^2^MOSff2 both are realized using sixteen transistors each. As a result, the area occupied by the proposed designs is significantly lesser than the conventional designs. Moreover, the number of clocked transistors in mC^2^MOSff1 is six as compared to eight in case of TGFF or conventional clocked inverter based flip-flop C^2^MOSff [23].

**Figure 7:**
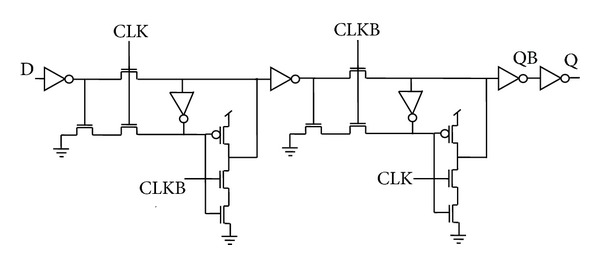
Schematic diagram for Write port master slave flip-flop (WPMS).

**Figure 8:**
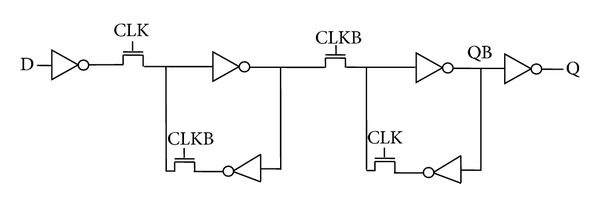
Schematic diagram for pass transistor logic style flip-flop (PTLFF).

**Figure 9:**
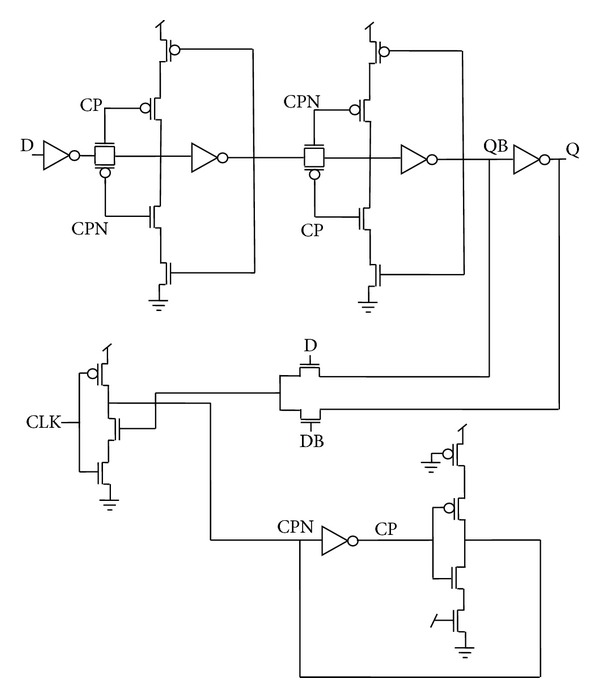
Schematic diagram for Gated master slave latch (GMSL).

**Figure 10:**
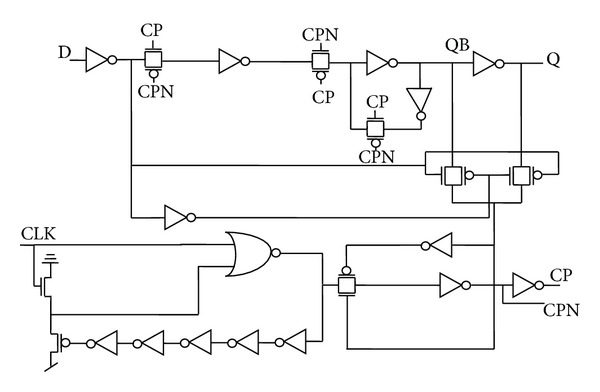
Schematic diagram for data transition look ahead flip-flop (DTLA).

To illustrate the superior performance of the proposed flip-flop configurations, other flip-flop topologies, namely, TGFF, WPMS, PTLFF, gated master-slave latch (GMSL) [10], and data transition look ahead flip-flop (DTLA) [11] belonging to the master-slave class have been used for comparisons. Out of the above mentioned topologies GMSL, and DTLA represent flip-flops with internal clock gating. Schematic diagrams of WPMS, PTLFF, GMSL and DTLA are shown in Figures 7, 8, 9, and 10, respectively.

## 3. Simulation Parameters, Test Bench, and Optimization Methodology

Table 1 lists the CMOS parameters used for creating the simulation environment. The flip-flops were designed to layout level in 180 nm/1.8 V CMOS process at 250 MHz clock frequency. The width of transistors in the feedback structures was invariably fixed at the minimum value 360 nm while the slope of the data and clock signals was kept at 100 ps. Performances of the various flip-flop configurations are evaluated through SPICE simulation of the circuits extracted from the layout with the inclusion of parasitics.

**Figure 11:**
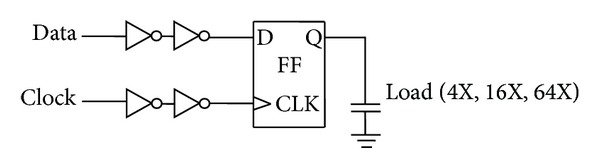
Simulation setup.

Figure 11 shows the simulation test bench for characterization and comparison of the FF designs [3]. The clock and data signals are fed to the flip-flop through a two stage buffer. Data-to-output delay (*T*_DQ,min⁡_) is used for performance comparisons. Logical effort theory is extensively used for designing fast CMOS circuits based on pencil and paper calculations and is widely adopted in the literature [24]. Hence, the delay sensitivity factor introduced by Alioto et al. [25] based on logical effort theory has been used for performance optimization.

A 16-cycle long pseudorandom sequence with a switching factor *α* = 0.5 is supplied at the data input for measurement of average power [26]. Since the delay and power characterization are strongly dependent on the capacitive load offered to FFs [27], varying capacitive loads {4, 16, 64}  *C*_min⁡_, where *C*_min⁡_ is the input capacitance of a symmetrical minimum inverter (*W*_*p*_ = 2*W*_*n*_ = 2*W*_min⁡_), have been used to test the FF behaviour. Transistor sizing methodology adopted is the same as that in [28, 29], whereas power-delay product (PDP) and power-delay-area product (PDAP) are the chosen figures of merit (FOM).

The expression relating the absolute gate capacitance (*C*_GATE_) in terms of fF (femtofarads) and absolute transistor width (*W*) in terms of nanometers (nm) obtained at 180 nm process node by fitting simulation data [30] is given as

(1) $$\begin{array}{c} C_{\text{GATE}}=\left(1.15\cdot 10^{-3}\right)\cdot W. \end{array}$$

LE method states that the optimized delay *D* of a path of *N* cascaded stages is

(2) $$\begin{array}{c} D=N\sqrt[{N}]{GBH}+P, \end{array}$$

(3) $$\begin{array}{c} D=N\sqrt[{N}]{F}+P, \end{array}$$

where *G*, *B*, *H* ( = *C*_*L*_/*C*_in_) are the logical effort, branching effort, and electrical effort while *P*, *F* (= *GBH*) and *C*_*L*_ are parasitic delay, path effort, and final load capacitance, respectively. One has the following:

(4) $$\begin{array}{c} D=P\left(1+t\right). \end{array}$$

From (2) and (4),

(5) $$\begin{array}{c} t=\frac{N\sqrt[{N}]{GB}\sqrt[{N}]{C_{L}}}{P\sqrt[{N}]{C_{\text{in}}}}, \end{array}$$

where *t* represents the relative delay increment with respect to parasitic delay. Equations (4) and (5) indicate that larger values of *C*_in_ lead to a saturation in the optimized delay and based on the above analysis, the delay sensitivity factor introduced by Alioto et al. [25] is utilized to obtain the upper bound on the transistor widths for exploration of the power-delay design space with least computational effort. Consider the following:

(6) $$\begin{array}{c} S_{D}^{C_{\text{in}}}=\frac{\partial D}{\partial C_{\text{in}}}\frac{C_{\text{in}}}{D}=-\frac{1}{N}\frac{t}{t+1}, \end{array}$$

where *S*_*D*_^*C*_in_^ is the delay sensitivity factor and is obtained from (3) to (5). The upper bounds on the normalized transistor widths *w*_*i*_ (normalized with respect to *W*_min⁡_) have been obtained such that the delay sensitivity remains under a minimum value *S*_min⁡_ which is chosen as −5% for our analysis. The input capacitance *C*_in_ of the flip-flop is expressed in terms of normalized width *w*1 as follows:

(7) $$\begin{array}{c} C_{\text{in}}=\left(w1\cdot 360+2\cdot w1\cdot 360\right)\left(1.15\cdot 10^{-3}\right). \end{array}$$

**Figure 12:**
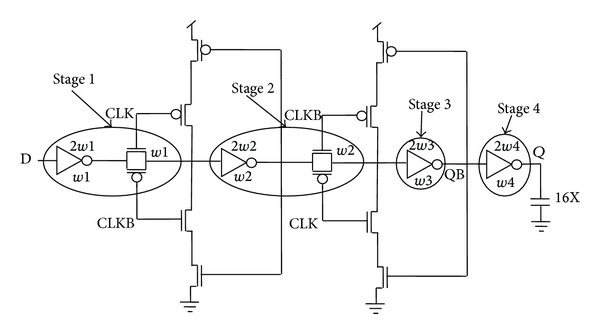
LE theory based transistor sizing methodology for transmission gate flip-flop.

Figure 12 shows the conventional TGFF design. The sizing is done by assuming the transistors in the critical path to be independent design variables (IDVs) and optimizing for maximum performance using LE theory. The inverter before transmission gate in the first stage protects the input terminal from noise variations [31].  Table 2 exhibits delay variation for increasing *C*_in_ values. It is noteworthy that the delay saturates at 153 ps for *C*_in_ = 24.8 fF. As a result, the upper bounds on transistor widths are exposed and the limits of power (energy)-delay design space are defined early in the design cycle [32]. The table also includes the corresponding power dissipation along with the power-delay product and it is observed that minimum power-delay product is obtained at *C*_in_ = 9.92 fF. The technology parameters used for capacitance calculations throughout this paper are listed in Table 3.

## 4. Results and Discussion

It is a well-established fact that the conventional C^2^MOS although slower, is skew tolerant and occupies lesser area than TGFF [23, 33]. Moreover, mC^2^MOSff1 and mC^2^MOSff2 show nearly identical characteristics in terms of power, delay, and area and hence only mC^2^MOSff1 is considered for comparisons.

**Figure 13:**
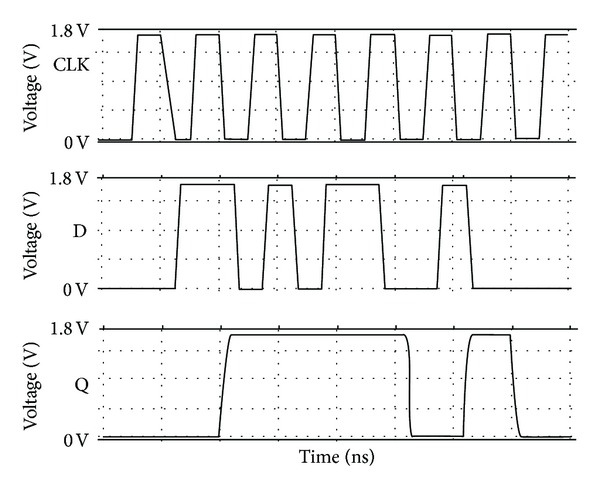
HSPICE simulation waveforms at 1 GHz clock frequency for mC^2^MOSff1.

**Figure 14:**
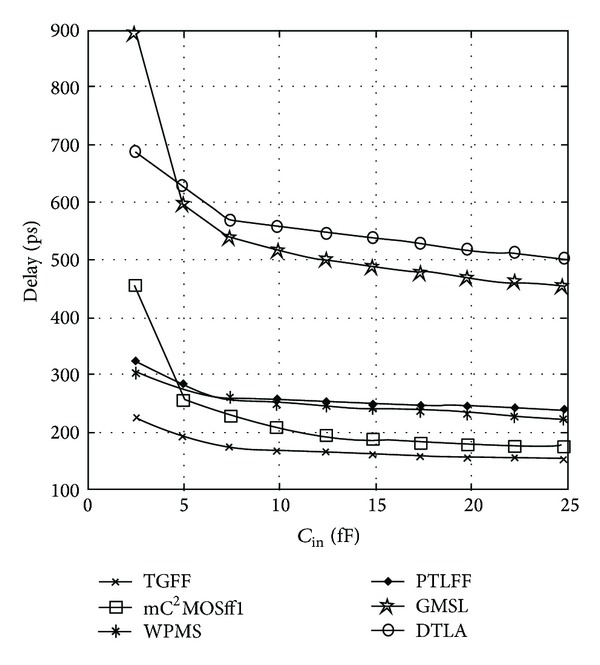
Variation in data-to-output delay with respect to FF input capacitance.

The waveforms in Figure 13 represent the transient analysis of mC^2^MOSFF1 carried out over a period of 8 clock cycles. The SPICE simulation results verify the correct flip-flop operation at 1 GHz clock frequency (all the flip-flops reported in the paper are designed for negative edge triggered operation). The variation of absolute data-to-output delays *T*_DQ,min⁡_ with FF input capacitance (*C*_in_) for 16X (19.92 fF) capacitive load is illustrated in Figure 14.

TGFF utilizes transmission gates in the critical path and hence it is faster than the rival designs. There is exactly the same number of stages in the critical path of TGFF and mC^2^MOSff1, the only difference being that the latching circuit in case of TGFF is an inverter followed by a clocked transmission gate (inverting latch), whereas a clocked/tristate inverter is present in mC^2^MOSff1. Logical effort of both the latches is considered to be two; however, it is apparent that an inverter followed by a transmission gate is faster because the output node is driven by both the transistors of the transmission gate in parallel and this behaviour is reflected in Figure 14. From the above discussion, it is obvious that the value of logical effort for an inverting latch can be assumed to be two for most theoretical purposes, but for comparison with a C^2^MOS latch, it must be slightly less than two if delays are to be modelled precisely.

Equation (2) clearly indicates that lesser branching effort leads to a faster circuit operation. The branching effort for a path with internal fan-out is expressed as [24]

(8) $$\begin{array}{c} b=\frac{C_{\text{on-path}}+C_{\text{off-path}}}{C_{\text{on-path}}}, \end{array}$$

where *C*_on-path_ represents the load capacitance along the path under analysis and *C*_off-path_ represents the capacitance of the connections that lead off the path.

The branching effort along the critical path is given as

(9) B=∏b.  i  

There are two branches each in TGFF and mC^2^MOSff1 represented as *b*1, *b*2 and *b*3, *b*4 in Figures 6 and 5(a), respectively. The branching effort corresponding to branches *b*1, *b*2, *b*3, and *b*4 is calculated as follows.

### 4.1. Branching Effort in Case of TGFF

One has the following.

 
*b*1 Calculation:
  (10) Con-path=Cgd(TN5)+Cdb(TN5) +Cgd(TP5)+Cdb(TP5)=8.43 fF,Coff-path=Cg(TN2)+Cg(TP2)=1.12 fF,
 
*b*1 = 1.13.
 
*b*2 Calculation:
  (11) $$\begin{array}{c} C_{\text{on-path}}=C_{g}\left(\text{TN}9\right)+C_{g}\left(\text{TP}9\right)=12.33\;\text{fF},\\ C_{\text{off-path}}=C_{g}\left(\text{TN}6\right)+C_{g}\left(\text{TP}6\right)=1.12\;\text{fF}, \end{array}$$
 
*b*2 = 1.09,
 
*B* = *b*1∗*b*2 = 1.23.

### 4.2. Branching Effort in Case of mC^2^MOSff1

One has the following.

 
*b*3 Calculation:
  (12) Con-path=Cg(TN14)+Cg(TP14)=7.76 fF,Coff-path=Cgd(TN16)+Cdb(TN16)+Cgd(TP16) +Cdb(TP16)=1.47 fF,
 
*b*3 = 1.18.
 
*b*4 Calculation:
  (13) $$\begin{array}{c} C_{\text{on-path}}=C_{g}\left(\text{TN}14\right)+C_{g}\left(\text{TP}14\right)=7.76\;\text{fF},\\ C_{\text{off-path}}=C_{g}\left(\text{TN}13\right)+C_{g}\left(\text{TP}13\right)=0.828\;\text{fF}, \end{array}$$
 
*b*4 = 1.10,
 
*B* = *b*3∗*b*4 = 1.30,

where *C*_*gd*_ is gate to drain capacitance, *C*_*db*_ is drain to body capacitance, and *C*_*g*_ is the gate capacitance of respective transistors.

Accordingly, using (2) and putting *G* = 4, *B* = 1.23, *H* = 19.92/12.4 = 1.60, *N* = 4, and *P* = 6, we have *D* = 12.7 (absolute delay 165.1 ps) for TGFF, whereas putting *G* = 4, *B* = 1.30, *H* = 19.92/12.4 = 1.60, *N* = 4, and *P* = 6, we have *D* = 12.79 (absolute delay 166.27 ps) for mC^2^MOSff1. Absolute delays *D*_abs_ are obtained by multiplying parameter *D* with parameter *τ* as follows:

(14) $$\begin{array}{c} D_{\text{abs}}=D\tau . \end{array}$$

It is clearly observed that the delay of mC^2^MOSff1 is marginally higher than the delay of TGFF. Now, keeping other parameters to be the same and assuming the logical effort of inverting latch to be 1.8, the updated value of TGFF is evaluated as *D* = 12.35 (absolute delay 160.55 ps).

The value of process dependent parameter *τ* is determined as approximately 13 ps using the calibration technique as mentioned by Sutherland et al. [24]. The detailed procedure is discussed in the Appendix. The absolute delay measurements obtained through simulation are 162 ps for TGFF and 196 ps for mC^2^MOSff1 which is in close agreement with the theoretical values 160.55 ps and 166.27 ps, respectively (typically within 15% error).

WPMS and PTLFF topologies show degraded performance due to the presence of pass transistors in the critical path while the speed of clock-gated structures is worst mainly because gating circuit is inserted between the clock and the flip-flop terminals which deteriorates the timing characteristics. The characterizations are done assuming that *C*_in_ = 12.4 fF and *C*_*L*_ = 19.92 fF (16X) where *C*_*L*_ represents the flip-flop load capacitance.

**Figure 15:**
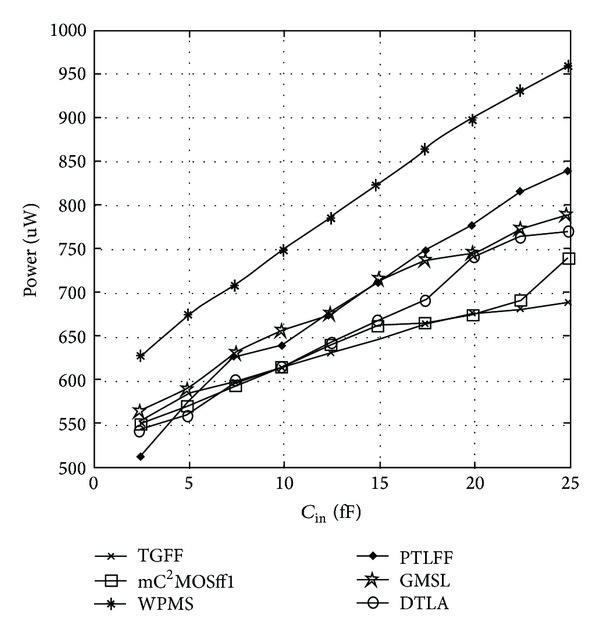
Variation in power dissipation as a function of FF input capacitance.

The variation of average power with *C*_in_ for 16X loading condition is depicted in Figure 15. Due to threshold voltage drop at internal nodes, WPMS and PTLFF display worst power dissipation characteristics because of short circuit power dissipation. GMSL and DTLA exhibit greater power dissipation than nongated counterparts because pseudorandom sequence has an activity factor of 0.5. The reason being the presence of additional comparator and clock gating circuit which is beneficial only at sufficiently low switching activities or otherwise leads to both increased area and power overhead.

### 4.3. Clock Load Calculations

One has the following.

TGFF:

(15) {Cg(TN1)+Cg(TP1)+Cg(TN5)+Cg(TP5)}   +{Cg(TN3)+Cg(TP3)+Cg(TN7)+Cg(TP7)}

{Transistors contributing towards clock load in the critical path} + {Transistors contributing towards clock load in the feedback structure}

 = 14.78 fF + 1.66 fF
 = 16.44 fF.

mC^2^MOSff1:

(16) {Cg(TN10)+Cg(TP10)+Cg(TN11)+Cg(TP11)}   +{Cg(TN16)+Cg(TP16)}

{Transistors contributing towards clock load in the critical path} + {Transistors contributing towards clock load in the feedback structure}

 = 22.18 fF + 0.84 fF
 = 23.02 fF.

Apart from the clock load, the capacitance value at internal nodes of mC^2^MOSff1 is reduced as compared to TGFF by eliminating transistors TN6 and TP6 from the feedback structure.

### 4.4. Capacitance Calculations at Internal Nodes of TGFF

*Internal Capacitance at Nodes P* *and K*

 Node P: *C*_*g*_(TN2) + *C*_*g*_(TP2) + *C*_*gd*_(TN5) + *C*_*db*_(TN5) + *C*_*gd*_(TP5) + *C*_*db*_(TP5) = 9.28 fF.
 Node K: *C*_*g*_(TN6) + *C*_*g*_(TP6) + *C*_*g*_(TN9) + *C*_*g*_(TP9) = 9.02 fF.

*Internal Capacitance at Nodes M* *and N*

 Node M: *C*_*gd*_(TN1) + *C*_*db*_(TN1) + *C*_*gd*_(TP1) + *C*_*db*_(TP1) + *C*_*fd*_(TN3) + *C*_*db*_(TN3) + *C*_*gd*_(TP3) + *C*_*db*_(TP3) + *C*_*g*_(TN4) + *C*_*g*_(TP4) = 18.41 fF.
 Node N: *C*_*gd*_(TN5) + *C*_*db*_(TN5) + *C*_*gd*_(TP5) + *C*_*db*_(TP5) + *C*_*gd*_(TN7) + *C*_*db*_(TN7) + *C*_*gd*_(TP7) + *C*_*db*_(TP7) + *C*_*g*_(TN8) + *C*_*g*_(TP8) = 14.80 fF.

### 4.5. Capacitance Calculations at Internal Nodes of mC^2^MOSff1

*Internal Capacitance at Nodes P' and K'*

 Node P': *C*_*g*_(TN12) + *C*_*g*_(TP12) = 9.76 fF.
 Node K': *C*_*g*_(TN13) + *C*_*g*_(TP13) + *C*_*g*_(TN14) + *C*_*g*_(TP14) + *C*_*gd*_(TN16) + *C*_*db*_(TP16) + *C*_*gd*_(TN16) + *C*_*db*_(TP16) = 10.06 fF.

*Internal Capacitance at Node M'*

 Node M': *C*_*g*_(TN15) + *C*_*g*_(TP15) = 12.35 fF.

**Figure 16:**
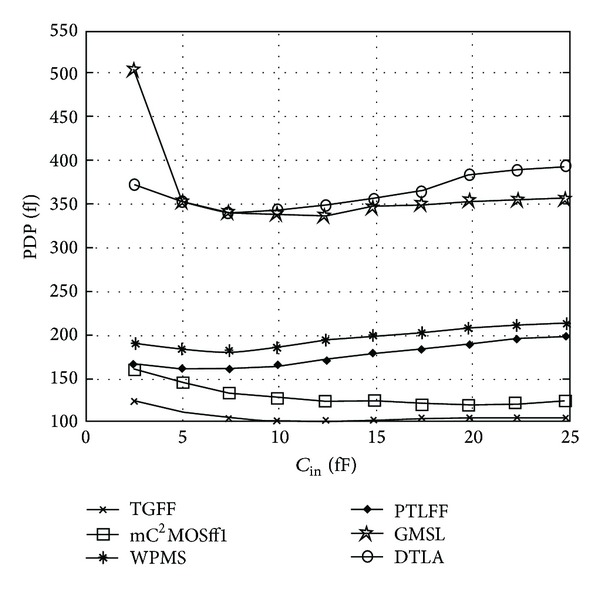
Power-delay product characteristics with varying FF input capacitance at 16X load.

It can be easily concluded from calculations above that a total of 19.34 fF capacitance has been reduced from the internal nodes in the critical path of mC^2^MOSff1 in comparison to TGFF. This leads to reduced internal power dissipation at these nodes as lesser capacitance has to be charged or discharged per clock cycle. However, reduction in the clock load of mC^2^MOSff1 due to transistors eliminated from the feedback structure is nullified due to PMOS transistors TP10 and TP11 whose size is twice that of transistors TP1 and TP5 in case of TGFF and as a result the total power dissipation of both the flip-flops is nearly the same as it can be clearly observed from Figure 16. Following a similar procedure, the clock load of various flip-flops is obtained and listed in Table 4 along with number of clocked transistors and power consumption values. It is seen that TGFF and mC^2^MOSff1 represent the most efficient designs in terms of reduced power consumption having power dissipation comparable to DTLA at *C*_in_ = 12.4 fF and *C*_*L*_ = 19.92 fF.

It can be observed that mC^2^MOSff1 has the least transistor count along with PTLFF while GMSL and DTLA consist of maximum number of transistors. Since only sixteen transistors are used for circuit realization of mC^2^MOSff1, power dissipation is comparable to TGFF. It is worth noting that GMSL and DTLA offer minimum clock load, as a result, these topologies exhibit least power dissipation at lower switching activities. The reason for extended clock-to-output delays of GMSL and DTLA is the insertion of clock gating circuitry while DTLA has a pulsed operation and hence shows negative set-up time requirements. Based on the power and delay measurements, power-delay product characteristics are derived for all the flip-flops as shown in Figure 16. The optimum power-delay product of gated structures GMSL and DTLA is, respectively, 3.30x and 3.34x times greater than optimum PDP of TGFF. Among the nonclock gated structures, pass transistors based designs WPMS and PTLFF exhibit 1.77x and 1.57x enhancement in the power-delay product with respect to the benchmark flip-flop TGFF. TGFF also shows 20% improvement over mC^2^MOSff1 in terms of minimum power-delay product. However, despite the fact that TGFF represents a better alternative in terms of performance and optimum power-delay product, the area requirements also remain a major concern. It has been observed in the literature that conventional C^2^MOS based flip-flop is up to 20–25% more efficient in terms of occupied chip area. This stems mainly from the fact that at layout level (i) in comparison to TGFF, diffusion areas of most of the transistors can be shared in C^2^MOS flip-flop [33], (ii) the number of contact holes can be reduced in the layout pattern [23], and (iii) less complicated feedback structure leads to fewer interconnections.

**Figure 17:**
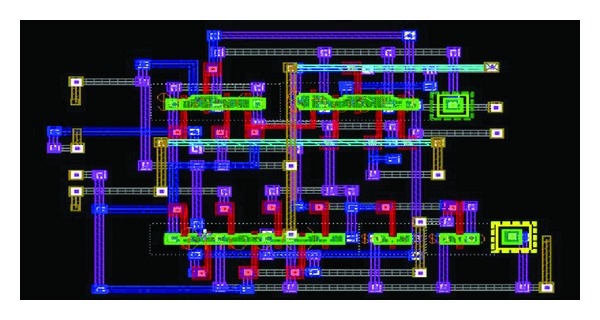
Layout implementation of TGFF.

**Figure 18:**
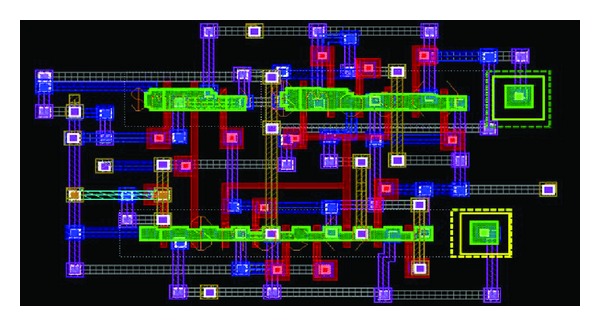
Layout implementation of mC^2^MOSff1.

The layouts were implemented using *C*_in_ = 12.4 fF, indicating almost similar transistor sizes throughout the critical path with the exception of TP10 and TP11 belonging to mC^2^MOSff1 which are twice in size compared to TP1 and TP5 in accordance with the LE theory. The layouts for TGFF and mC^2^MOSff1 are shown in Figures 17 and 18, respectively.  Table 5 clearly shows that while TGFF is better in terms of PDP by 18.4%, mC^2^MOSff1 shows a 12.4% improvement in the PDAP making it suitable for high density applications where performance can be compromised.

**Figure 19:**
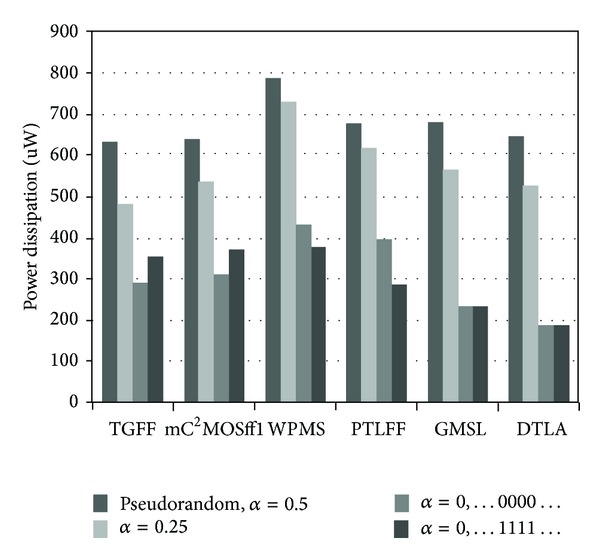
Comparison chart of power dissipation at different switching frequencies.

The power dissipation results as illustrated in Figure 19 are obtained using *C*_in_ = 12.4 fF which ensures that all the transistors in the critical path have similar widths. At zero switching activity, clock-gated topologies are the most power efficient. GMSL and DTLA show GMSL 32.5% and 46.3% reduction in power in case of logic high at the input, whereas for logic low, the power consumption is reduced by 19.2% and 35.4%, respectively. Again, it can be clearly observed that there is only a slight difference in the power dissipation of TGFF and mC^2^MOSff1 at different switching activities.

The correct functionality of the proposed flip-flop mC^2^MOSff1 is validated by designing an 8-bit ripple counter at 16X capacitive load and the average power measurements were carried out over 256 clock cycles. It was noticed that the power consumption of the mC^2^MOSff1 based counter is comparable to the TGFF at varying frequencies. Again, LE theory has been adopted for sizing individual flip-flops in each counter for optimum performance which is expressed in detail in the Appendix.

The flip-flops were also designed and simulated to layout level with inclusion of parasitics at 130 nm, 90 nm, and 65 nm CMOS processes to address scalability issues at more advanced process nodes. The simulation test bench and optimization methodology are similar as mentioned in Section 3. PVT variations are emphasized to evaluate the performance of flip-flops at all process corners, namely, FF, SS, FS, and SF with voltages scaled from 0.9 to 1.1 V while the temperatures varied from 0 to 125 degrees as shown in Table 6. The simulation and technology parameters are also listed in Table 6 where *C*_*G*_ represents the capacitance per unit gate oxide and was evaluated to be 1.3 fF/um by fitting simulation data. In addition, the capacitances per unit length of poly, metal 1 and metal 2 interconnects are also mentioned.

**Figure 20:**
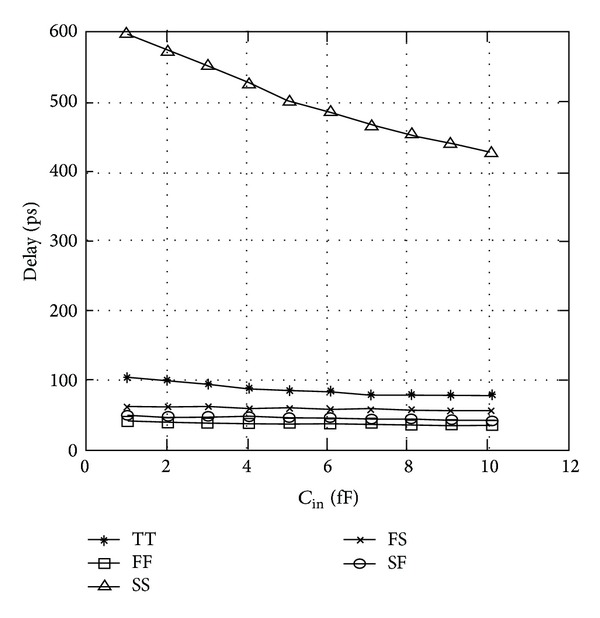
Delay variations for mC^2^MOSff1 at 16X loading for different process corners.

**Figure 21:**
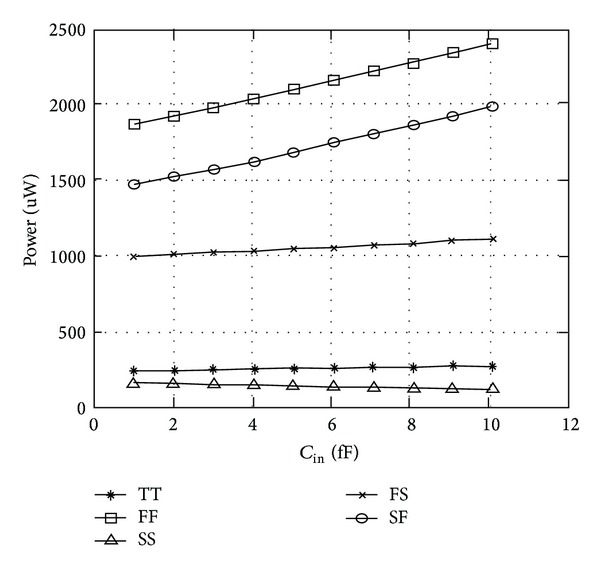
Delay variations for mC^2^MOSff1 at 16X loading for different process corners.

For illustration purposes, the delay and power variations with the flip-flop input capacitance with respect to different process corners at 65 nm CMOS technology for mC^2^MOSff1 are demonstrated in Figures 20 and 21, respectively, at 16X capacitive loading. Both mC^2^MOSff1 and mC^2^MOSff2 showed correct circuital behaviour at the aforementioned process nodes which indicates that no internal noise violations exist especially due to the fact that logic levels are retained even at FF process corner. However, it is to be pointed out that mC^2^MOSff1 in a manner similar to TGFF starts to fail at SS corner for lower values of *C*_in_ [34].

## 5. Conclusion

In this paper, an alternative architecture for designing C^2^MOS based flip-flops is presented with a modified feedback strategy while preserving the fully static operation. Using the new feedback approach, a modified topology mC^2^MOSff1 is proposed with decreased parasitic capacitances at internal nodes in comparison to the TGFF which is the finest design in terms of PDP. However, postlayout simulations and analyses indicate that the modified configuration mC^2^MOSff1 presents the best alternative in terms of PDAP among all the conventional designs. Therefore, for high performance applications, TGFF still remains the best choice but it can be replaced by mC^2^MOSff1 for high density applications. Comparisons were carried out with state-of-the-art flip-flops in the master-slave class. The simulation results are well supported with mathematical analysis based on logical effort theory within acceptable error (typically less than 15%).

## Figures and Tables

**Figure 22:**
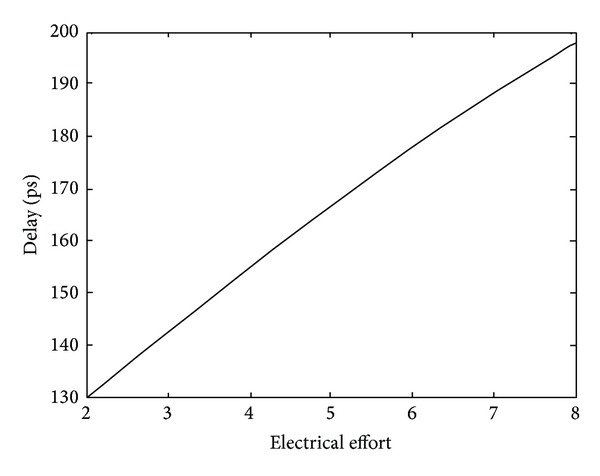
Delay versus fanout curve for an inverter at 180 nm/1.8 V CMOS process.

**Figure 23:**
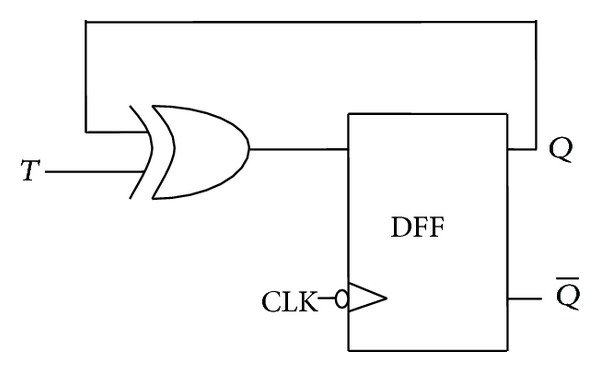
Conversion of D flip-flop to T flip-flop.

**Figure 24:**
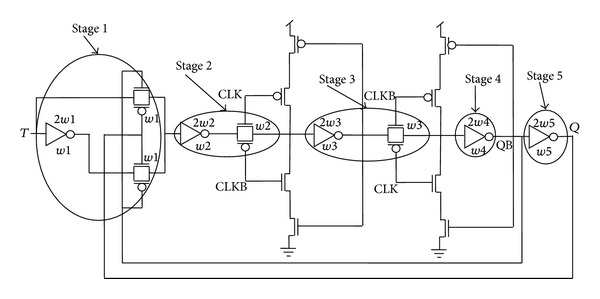
TGFF based T flip-flop.

**Figure 25:**
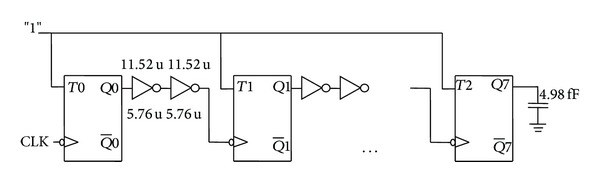
Schematic diagram of a modulo 256 ripple counter with intermediate buffers.

**Table 1:** Simulation parameters.

Parameter	Value
*W*_min⁡_	360 nm
*L*_min⁡_	140 nm
*C*_min⁡_	1.24 fF
*V*_DD_	1.8 V
Frequency	250 MHz
Signal slope	100 ps

**Table 2:** Traditional transmission gate flip-flop at 19.92 fF load (16X).

*C*_in_ (fF)	*w*1	*w*2	*w*3	*w*4	*T*_DQ, min_ (ps)	Power (uW)	PDP (fJ)
2.48	2	2.35	2.79	6.65	226	554	125.2
4.96	4	3.95	3.95	7.91	191	585	111.7
7.44	6	5.35	4.84	8.76	173	599	103.6
9.92	8	6.65	5.59	9.41	166	615	102
12.4	10	7.86	6.25	9.95	162	632	102.3
14.8	12	9.01	6.85	10.4	159	648	103
17.3	14	10.1	7.40	10.8	157	665	104.4
19.8	16	11.1	7.91	11.2	155	675	104.6
22.3	18	12.2	8.39	11.5	154	682	105
24.8	20	13.2	8.84	11.8	153	689	105.4

**Table 3:** Technology parameters used for estimation of capacitances.

Parameter	*C*_gdo_ (F/m)	*C*_gso_ (F/m)	*C*_jsw_ (F/m)	*C*_*j*_ (F/m^2^)	*L*_*D*_ (m)	*L*_*S*_ (m)
NMOS	2.78*E* − 10	2.78*E* − 10	7.9*E* − 10	0.00365	31.6*E* − 09	31.6*E* − 09
PMOS	2.78*E* − 10	2.78*E* − 10	1.44*E* − 9	0.00138	31.6*E* − 09	31.6*E* − 09

**Table 4:** Comparison of flip-flop parameters at *C*_in_ = 12.4 fF and 16X capacitive loading.

Design	TGFF	mC^2^MOSff1	WPMS	PTLFF	GMSL	DTLA
Transistor count	20	16	24	16	31	46
No. of clocked transistors	8	6	6	4	2	3
Clock-to-output delay (ps)	92	116	206	204	419	683
Optimum setup time (ps)	70	80	40	50	80	−140
Hold time (ps)	−19	−21	−33	−32	−23	25
*T*_DQ, min_ (ps)	162	196	246	254	499	543
Clock load (fF)	16.44	23.02	9.05	8.22	7.76	7.31
Power dissipation (uW)*	632	640	786	679	676	643
Leakage Power (uW)	59.38	57.51	72.64	69.83	74.91	76.73

*Pseudorandom sequence with *α* = 0.5 is used for power calculations.

**Table 5:** PDAP comparison of TGFF and mC^2^MOSff1.

Design	Transistor count	Transistor widths (um)	Delay (ps)	Power (uW)	Layout area (um^2^)	PDP (fJ)	PDAP (fJ·um^2^)
TGFF	20	52.52	162	632	175	102.3	17902
mC^2^MOSff1	16	58.95	196	640	125	125.4	15675

**Table 6:** Flip-flop simulation parameters at 65 nm CMOS technology.

Process corner	Temperature (°C)	*V*_DD_	Simulation/technology parameters				
TT	70	1	*L*_min⁡_	*W*_min⁡_	*C*_min⁡_	Frequency	Signal slope
FF	0	1.1	60 nm	120 nm	507 aF	2 GHz	20 ps
SS	125	0.9					
FS	70	1	*C*_Poly_ = 0.268 *C*_*G*_		*C*_metal1_ = 0.215 *C*_*G*_		*C*_metal2_ = 0.175 *C*_*G*_
SF	70	1					

## Appendices

### A. Delay Calibration Using LE Theory

For modelling delays using LE theory initially, all the delays are expressed in terms of a basic delay unit *τ* which is process dependent such that the absolute delay is represented as the product of a unit less delay of the gate as shown in (2), and the delay unit *τ*. Accordingly,

(A.1) $$\begin{array}{c} D_{\text{abs}}=D\tau . \end{array}$$

While *D* represents the delay for a multistage path, *d* corresponds to the delay of a single stage logic gate. Parameter *τ* needs to be estimated in order to obtain absolute delays and accordingly a delay versus fanout curve is determined for an inverter as shown in Figure 22 by fitting simulation data. The curve is approximated as a straight line and the slope of the line represents *τ* since *d* = (*gh* + *p*)*τ* and logical effort of an inverter is 1. In our case, *τ* is estimated as 13 ps.

### B. Implementation of 8-Bit Ripple Counter

An 8-bit asynchronous counter was implemented by converting the D flip-flop configuration to a T flip-flop configuration using an EXOR gate as illustrated in Figure 23.

The T flip-flop designed using TGFF is shown in Figure 24. It is considered to be a five stage design and optimized for highest speed using LE theory. The EXOR gate was realized using transmission gates as revealed in Stage 1 of Figure 24. A similar procedure was followed for designing mC^2^MOSff1 based T flip-flop.

For designing the modulo 256 counter, the output *Q* of each stage is connected to the clock terminal of the next stage through two intermediate inverters (acting as a buffer) sized (*W*_*p*_ = 11.52 u, *W*_*n*_ = 5.76 u) such that the input capacitance of the first inverter acts as the load capacitance for the flip-flop configuration of the previous stage as depicted in Figure 25. As a result, the load at the output terminal of each flip-flop is uniformly fixed at 19.92 fF.

## References

[1] Kawaguchi H, Sakurai T (1998). A reduced clock-swing flip-flop (RCSFF) for 63% power reduction. *IEEE Journal of Solid-State Circuits*.

[2] Yeap G (1998). *Practical Low Power Digital VLSI Design*.

[3] Oklobdzija V, Stojanovic V, Markovic D, Nedovic N (2003). *Digital System Clocking: High-Performance and Low-Power Aspects*.

[4] Mesgarzadeh B, Hansson M, Alvandpour A (2007). Jitter characteristic in charge recovery resonant clock distribution. *IEEE Journal of Solid-State Circuits*.

[5] Giacomotto C, Nedovic N, Oklobdzija VG (2007). The effect of the system specification on the optimal selection of clocked storage elements. *IEEE Journal of Solid-State Circuits*.

[6] Gerosa G, Gary S, Dietz C (1994). 2.2 W, 80 MHz superscalar RISC microprocessor. *IEEE Journal of Solid-State Circuits*.

[7] Markovic D, Tschanz J, De V (2003). Transmission-gate based flip-flop. *US Patent 6642765*.

[8] Hsu SK, Mathew SK, Anders MA (2006). A 110 GOPS/W 16-bit multiplier and reconfigurable PLA loop in 90-nm CMOS. *IEEE Journal of Solid-State Circuits*.

[9] Hossain R, Wronski LD, Albicki A (1994). Low power design using double edge triggered flip-flops. *IEEE Transactions on Very Large Scale Integration (VLSI) Systems*.

[10] Strollo AGM, Napoli E, de Caro D (2001). Low-power flip-flops with reliable clock gating. *Microelectronics Journal*.

[11] Nogawa M, Ohtomo Y (1998). A data-transition look-ahead DFF circuit for statistical reduction in power consumption. *IEEE Journal of Solid-State Circuits*.

[12] Klass F, Amir C, Das A (1999). A new family of semidynamic and dynamic flip-flops with embedded logic for high-performance processors. *IEEE Journal of Solid-State Circuits*.

[13] Zhao P, Darwish T, Bayoumi M Low power and high speed explicit-pulsed flip-flops.

[14] Partovi H, Burd R, Salim U, Weber F, DiGregorio L, Draper D Flow-through latch and edge-triggered flip-flop hybrid elements.

[15] Heald R, Aingaran K, Amir C (2000). Third-generation SPARC V9 64-b microprocessor. *IEEE Journal of Solid-State Circuits*.

[16] Nedovic N, Aleksic M, Oklobdzija VG Conditional techniques for low power consumption flip-flops.

[17] Naffziger SD, Colon-Bonet G, Fischer T, Riedlinger R, Sullivan TJ, Grutkowski T (2002). The implementation of the itanium 2 microprocessor. *IEEE Journal of Solid-State Circuits*.

[18] Kong B-S, Kim S-S, Jun Y-H (2001). Conditional-capture flip-flop for statistical power reduction. *IEEE Journal of Solid-State Circuits*.

[19] Shin S, Kong B (2005). Variable sampling window flip-flops for low power high-speed VLSI. *IEE Proceedings of Circuits, Devices and Systems*.

[20] Nikolić B, Oklobdžija VG, Stojanovič V, Jia W, Chiu JK-S, Leung MM-T (2000). Improved sense-amplifier-based flip-flop: design and measurements. *IEEE Journal of Solid-State Circuits*.

[21] Nedovic N, Oklobdzija VG, Walker WW A clock skew absorbing flip-flop.

[22] Strollo AGM, de Caro D (2000). Low power flip-flop with clock gating on master and slave latches. *Electronics Letters*.

[23] Suzuki Y, Odagawa K, Abe T (1973). Clocked CMOS Calculator Circuitry. *IEEE Journal of Solid-State Circuits*.

[24] Sutherland I, Sproull B, Harris D (1998). *Logical Effort: Designing Fast CMOS Circuits*.

[25] Alioto M, Consoli E, Palumbo G (2010). General strategies to design nanometer flip-flops in the energy-delay space. *IEEE Transactions on Circuits and Systems I*.

[26] Stojanovic V, Oklobdzija VG (1999). Comparative analysis of master-slave latches and flip-flops for high-performance and low-power systems. *IEEE Journal of Solid-State Circuits*.

[27] Heo S, Asanovic K Load-sensitive flip-flop characterization.

[28] Alioto M, Consoli E, Palumbo G (2011). Analysis and comparison in the energy-delay-area domain of nanometer CMOS Flip-Flops. Part I: methodology and design strategies. *IEEE Transactions on Very Large Scale Integration (VLSI) Systems*.

[29] Alioto M, Consoli E, Palumbo G (2011). Analysis and comparison in the energy-delay-area domain of nanometer CMOS Flip-Flops. Part II: results and figures of merit. *IEEE Transactions on Very Large Scale Integration (VLSI) Systems*.

[30] Palumbo G, Pennisi M Design guidelines for high-speed transmission-gate latches: analysis and comparison.

[31] Consoli E, Palumbo G, Pennisi M (2012). Reconsidering high-speed design criteria for transmission-gate-based master-slave flip-flops. *IEEE Transactions on Very Large Scale Integration (VLSI) Systems*.

[32] Alioto M, Consoli E, Palumbo G (2012). From energy-delay metrics to constraints on the design of digital circuits. *International Journal of Circuit Theory and Applications*.

[33] Chao HJ, Johnston CA (1989). Behavior analysis of CMOS D flip-flops. *IEEE Journal of Solid-State Circuits*.

[34] Dao HQ, Nowka K, Oklobdzija VG Analysis of clocked timing elements for dynamic voltage scaling effects over process parameter variation.

